# The Papain-like Cysteine Protease *HpXBCP3* from *Haematococcus pluvialis* Involved in the Regulation of Growth, Salt Stress Tolerance and Chlorophyll Synthesis in Microalgae

**DOI:** 10.3390/ijms222111539

**Published:** 2021-10-26

**Authors:** Wenfu Liu, Chunli Guo, Danqiong Huang, Hui Li, Chaogang Wang

**Affiliations:** Shenzhen Key Laboratory of Marine Bioresource and Eco-Environmental Science, Shenzhen Engineering Laboratory for Marine Algal Biotechnology, Guangdong Provincial Key Laboratory for Plant Epigenetics, College of Life Sciences and Oceanography, Shenzhen University, Shenzhen 518060, China; 2170257312@email.szu.edu.cn (W.L.); lcg521014@126.com (C.G.); dqhuang@szu.edu.cn (D.H.); lihui80@szu.edu.cn (H.L.)

**Keywords:** *Haematococcus pluvialis*, *HpXBCP3*, *Chlamydomonas reinhardtii*, salt stress sensitivity, chlorophyll synthesis, cell proliferation

## Abstract

The papain-like cysteine proteases (PLCPs), the most important group of cysteine proteases, have been reported to participate in the regulation of growth, senescence, and abiotic stresses in plants. However, the functions of PLCPs and their roles in stress response in microalgae was rarely reported. The responses to different abiotic stresses in *Haematococcus pluvialis* were often observed, including growth regulation and astaxanthin accumulation. In this study, the cDNA of *HpXBCP3* containing 1515 bp open reading frame (ORF) was firstly cloned from *H. pluvialis* by RT-PCR. The analysis of protein domains and molecular evolution showed that *HpXBCP3* was closely related to *AtXBCP3* from *Arabidopsis*. The expression pattern analysis revealed that it significantly responds to NaCl stress in *H. pluvialis*. Subsequently, transformants expressing *HpXBCP3* in *Chlamydomonas reinhardtii* were obtained and subjected to transcriptomic analysis. Results showed that *HpXBCP3* might affect the cell cycle regulation and DNA replication in transgenic *Chlamydomonas*, resulting in abnormal growth of transformants. Moreover, the expression of *HpXBCP3* might increase the sensitivity to NaCl stress by regulating ubiquitin and the expression of WD40 proteins in microalgae. Furthermore, the expression of *HpXBCP3* might improve chlorophyll content by up-regulating the expression of NADH-dependent glutamate synthases in *C. reinhardtii*. This study indicated for the first time that *HpXBCP3* was involved in the regulation of cell growth, salt stress response, and chlorophyll synthesis in microalgae. Results in this study might enrich the understanding of PLCPs in microalgae and provide a novel perspective for studying the mechanism of environmental stress responses in *H. pluvialis*.

## 1. Introduction

In plants and microalgae, proteases play essential roles in organism growth, reproduction, development, photosynthesis, senescence, and environmental stress responses [1,2]. For example, in *Chlamydomonas reinhardtii*, proteases are involved in the cell division [3]. In higher plants, papain-like cysteine proteases (PLCPs) are one of the major catalytic classes of protease [1]. According to the protein structure, *Arabidopsis* PLCPs are divided into nine subgroups [4], PLCPs are produced in the form of inactive precursors containing a signal peptide for protein secretion, an autoinhibitory prodomain for removal after maturation, and an active protease domain with the catalytic triad Cys-His-Asn [4,5,6]. Some members of PLCPs also contain a plant C-terminal granulin domain [6].

Studies showed that some PLCPs, such as *AtXCP1* and *AtSAG12*, were involved in dwarfism and early leaf senescence in plants [5]. In *Arabidopsis thaliana*, the PLCP gene *AtCEP1* was essential for the normal development of germ cells during xylem cell development [7]. Additionally, inhibition of the PLCPs activity reduced the content of chlorophyll and carotenoid by affecting the morphology and the function of chloroplasts in plants [8]. Similarly, in pepper and barley, the inhibition of PLCPs activity could lead to the enhanced tolerance to salt and osmotic stresses [9,10]. It showed that PLCPs from plants play different roles in growth, development, cell apoptosis, senescence, biotic and abiotic stresses [5,7,11].

Recent studies confirmed that PLCPs from plants involved in stress response. Even though *H. pluvialis* greatly responded to different stresses, there were few reports about the sequences of PLCPs or the functions of PLCPs in *H. pluvialis*. In this study, a PLCP gene from *H. pluvialis* was cloned and named as *HpXBCP3* based on its high sequence similarity with *AtXBCP3*. Its transcription profiles responding to NaCl stress were studied. Furthermore, *HpXBCP3* was introduced into *C. reinhardtii* to evaluate its functions. This is the first report regarding the cloning, expressing, and functional identification of the PLCP gene in microalgae. Results in this study confirmed that the *HpXBCP3* from *H. pluvialis* participated in some important biological processes such as growth, stress tolerance, and chlorophyll synthesis. It would help to understand the functions of PLCPs in microalgae and provide a new insight into the mechanism of environmental stress responses in *H. pluvialis*.

## 2. Results

### 2.1. Cloning and Sequence Analysis of HpXBCP3 from H. pluvialis

**Figure 1:**
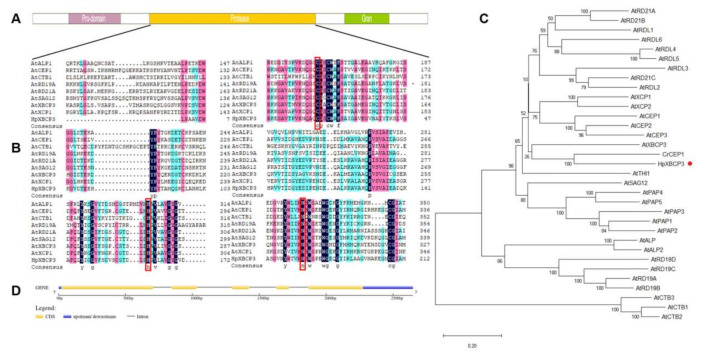
Sequence analysis of *HpXBCP3* gene. (**A**) Distribution of primary domains on *HpXBCP3*. (**B**) Alignment of the deduced amino acids of protease domain of *HpXBCP3* and *A. thaliana* PLCPs. The red box means the active protease domain contains the catalytic triad Cys-His-Asn. (**C**) Neighbor-joining phylogenetic tree based on nucleotide sequences of *HpXBCP3*, *CrCEP1* from *C. reinhardtii*, and PLCPs from *A. thaliana*. (**D**) Schematic diagram of *HpXBCP3* gene structure, which was predicted by aligning with corresponding genome sequences.

Based on a transcriptome data of *H. pluvialis*, one candidate of PLCP gene annotated as cysteine protease was found and successfully isolated. The sequence of this candidate gene was composed of 1533 bp and contained a 1515 bp open-reading frame (ORF) encoding a putative protein of 504 amino acids. The protein domain analysis found that it contained the autoinhibitory prodomain, the protease domain, and the granulin domain (Figure 1A), which is similar as *AtRD21A* belonging to PLCP subfamily 1 and *AtXBCP3* belonging to PLCP subfamily 4 in *Arabidopsis*. Aligning the protease domain with eight representative PLCP genes from *A. thaliana*, it showed that the protease domain was closer to XCP2 and XCP1 which suggested that they might have similar function. The conserved catalytic triad amino acids were also found in the protease domain (Figure 1B, Table S1). Additionally, the phylogenetic tree analysis showed that the PLCP gene from *H. pluvialis* belonged to one sub-clade, together with *CrCEP1* from *C. reinhardtii*. Furthermore, amino acid sequences of *HpXBCP3* and *CrCEP1* were close to *AtXBCP3* (Figure 1C, Table S1). Hence, the PLCP gene from *H. pluvialis* was named as *HpXBCP3* according to the homology of protease domains and phylogenetic tree.

Furthermore, the genomic sequence of *HpXBCP3* was obtained by Blastn against *H. pluvialis* genome sequence. By aligning the genome sequence and cDNA nucleotides, it revealed that *HpXBCP3* owned five exons and four introns (Figure 1D). Based on prediction using online software such as YLOC and Plant-Ploc, *HpXBCP3* could be located in the cytoplasm (Table 1).

### 2.2. HpXBCP3 Responded to NaCl Stress

**Figure 2:**
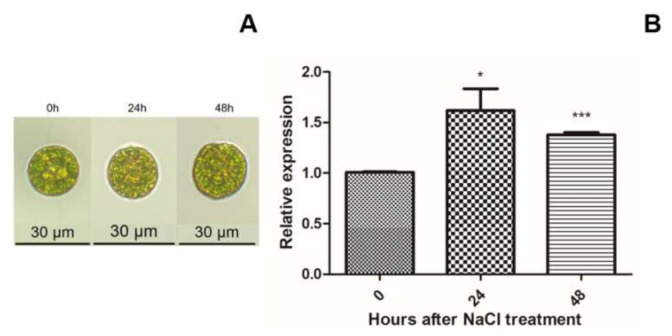
Morphologic and molecular responses of *H. pluvialis* to NaCl stress. (**A**) The representative morphology of *H. pluvialis* cells under microscope at different time points under NaCl stress. (**B**) Relative expression level of *HpXBCP3* responding to NaCl stress. Data shown were mean ± SD from three independent experiments. ***: significance the level of *p* < 0.001, *: significance at the level of *p* < 0.05.

When treated with 86 mM NaCl, the thickening of cell wall and the flagellum abscission was observed in *H. pluvialis* after 24 h, while astaxanthin accumulation was almost invisible (Figure 2A). In order to find out whether *HpXBCP3* was involved in the NaCl stress responses, its transcriptional activity was detected by qRT-PCR. Results showed the expression of *HpXBCP3* was obviously increased at 24 h and 48 h (Figure 2B) after NaCl stress treatment, suggesting that *HpXBCP3* was responsive to NaCl stress.

### 2.3. Expressing HpXBCP3 in C. reinhardtii

**Figure 3:**
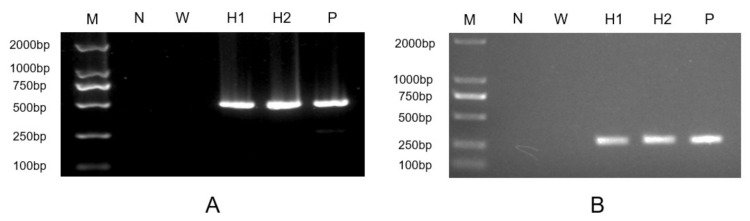
Genomic PCR and RT-PCR analysis of the *HpXBCP3* gene in transgenic *Chlamydomonas*. (**A**) Genomic PCR analysis of *HpXBCP3* gene. (**B**) RT-PCR analysis of *HpXBCP3* gene. N: negative control using ddH_2_O as template; P: positive control using plasmid DNA of pDb-124-HpXBCP3 as template; W: wild type of *C. reinhardtii* cc849; H1 and H2: transgenic *Chlamydomonas* as representative. M: DL 2000 DNA ladder marker.

To study the functions of *HpXBCP3*, the pDb124-HpXBCP3 plasmid was introduced into *C. reinhardtii* cc849 using the glass-bead method. The green colonies were visible after two weeks and then transferred to the new TAP agar medium containing 10 μg·mL^−1^ zeocin and 100 μg·mL^−1^ ampicillin. The PCR results showed that the foreign gene *HpXBCP3* was successfully inserted into *C. reinhardtii* genome (Figure 3A). The *HpXBCP3* was under the control of *PsaD* 5′ fused promoter, which could ensure its expression in *C. reinhardtii*. Subsequently, RT-PCR results showed the presence of the target gene in transformants and sequencing revealed that the nucleotides of amplified fragment were the same as expected (Figure 3B). These results confirmed that *HpXBCP3* was expressed in transgenic *Chlamydomonas* overexpressor lines which were named OEH.

### 2.4. Physiological Changes of Transgenic C. reinhardtii Expressing HpXBCP3

**Figure 4:**
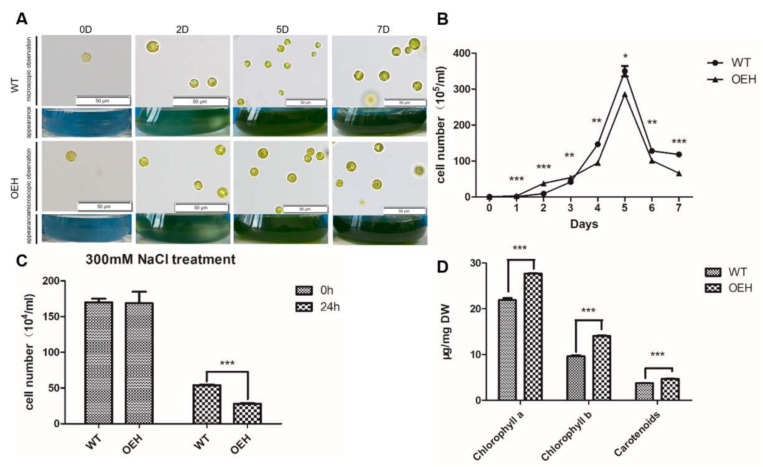
Physiological Changes of Transgenic *C. reinhardtii* Expressing *HpXBCP3***.** (**A**) Appearance and microscopic observation of *C. reinhardtii* (WT and OEH). (**B**) The growth curve of wild type and transgenic *Chlamydomonas*. WT: wild type of *C. reinhardtii* cc849. OEH: transgenic *Chlamydomonas* expressing *HpXBCP3*. Data shown were mean ± SD from three independent experiments. (**C**) The cell density of OEH and wide type of *Chlamydomonas* under NaCl stress. WT, the wild type of *C. reinhardtii* cc849. OEH, transgenic *Chlamydomonas*. Data shown were mean ± SD from three independent experiments. (**D**) Changes of chlorophyll a/b and carotenoids contents in OEH and wide type of *Chlamydomonas*. WT, the wild type of *C. reinhardtii* cc849. OEH, transgenic *Chlamydomonas*. Data shown were mean ± SD from four independent experiments. ***, **, and * indicates the statistical significance at the level of *p* < 0.001, *p* < 0.01, and *p* < 0.05, respectively.

Even though the morphology was no difference between the OEH and wild type of *C. reinhardtii* cc849 (WT) grown under normal culture conditions (Figure 4A), their growth curves were different (Figure 4B). From day 1 to day 3, the growth of OEH was faster than WT. Especially in day 2, the cell density of WT was only 23.5% of OEH. However, the growth of OEH was slowed down and the cell density was even lower than WT in day 4. The cell density of WT and OEH reached peak at day 5, whereas the WT had 18.1% higher cell density than OEH. In day 7, the cell density of WT was 77.5% higher than that of OEH. Overall, the trend of growth curve of OEH was the same as that of WT. Therefore, the expression of *HpXBCP3* in *C. reinhardtii* might increase cell growth at the early phase of culture, but decrease cell growth later.

The OEH and WT were treated with 300 mM NaCl for 24 h to evaluate their performance under NaCl stress. The cell density was significantly decreased in both WT and OEH after treatment. It is interesting that the cell density of WT was higher, which was almost double of that in OEH (Figure 4C), indicating that *HpXBCP3* increased the sensitivity of OEH to NaCl stress.

Literatures reported that the deletion or over-expression of PLCPs genes caused the expression change of genes related to chlorophyll metabolism. Here, it was found that the content of chlorophyll a/b and carotenoids in OEH were significantly higher than that in WT (Figure 4D). Specifically, the content of chlorophyll a in OEH was 26.5% higher than that in WT. Even more, that for the chlorophyll b and carotenoids was 48.4% and 25.9%, respectively.

### 2.5. Transcriptomic Analysis of OEH

**Figure 5:**
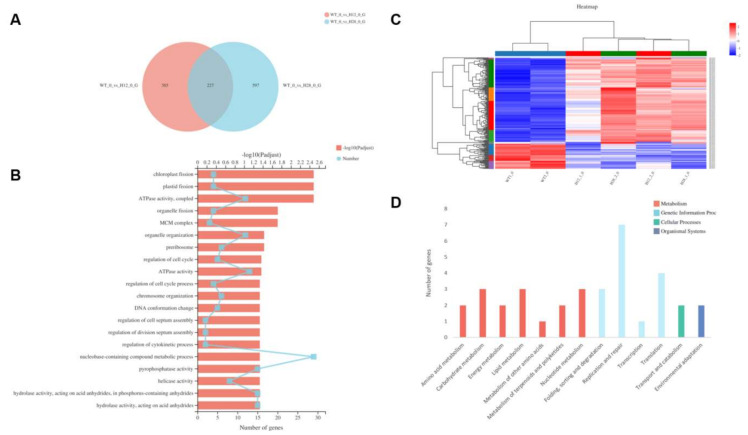
Transcriptome analysis of OEH. (**A**) The Venn diagram of differentially expressed genes. WT, the wild type of *C. reinhardtii* cc849. H12 and H28, transgenic *Chlamydomonas*. (**B**) Top 20 gene function classification of DEGs annotated by Gene ontology (GO). (**C**) Clustering displays of expression ratios (H12 or H28 vs. the wild type) of DEGs. Red color indicates up-regulation and blue denotes down-regulation. The rectangles with different colors on the left were represented in turn as cubcluster 9 (contained 1 gene), 8 (contained 3 genes), 2 (contained 57 genes), 4 (contained 27 genes), 1 (contained 60 genes), 5 (contained 24 genes), 10 (contained 4 genes), 3 (contained 23 genes), 6 (contained 12 genes) and 7 (contained 16 genes) from top to bottom. WT, the wild type of *C. reinhardtii* cc849. H12 and H28, transgenic *Chlamydomonas*. (**D**) KEGG pathway classification of DEGs. The X axis presents specific pathways in the second hierarchy. The different colors in histogram represent four functional categories of pathways in the first hierarchy.

The transcriptome of two individual OEH (H12 and H28) and WT grew under normal conditions were compared in this study. Overall, compared with WT, 613 differentially expressed genes (DEGs) in H12 and 825 DEGs in H28 were found, respectively, and they shared 227 DEGs in two compared groups (Figure 5A). Among them, eight DEGs were selected based on their high fold change and/ or functional relevance as representatives to evaluate the accuracy of transcriptomic data. The results of qRT-PCR revealed that their expression patterns were closely agreed with the transcriptomic data (Table S2).

#### 2.5.1. GO Significant Enrichment Analysis of DEGs

Based on the GO significant enrichment analysis, the 227 DEGs shared between WT and OEH belonged to 20 groups. Among them, 29 genes were in GO term “nucleobase-containing compound metabolic process”, which had the most genes. There were 22 genes involved in DNA replication and cell proliferation, such as “minichromosome maintenance complex (MCM complex)”, “regulation of cell cycle”, and “helicase activity”. Four genes were related to cell division, such as “chloroplast fission”, “plastid fission”, and “organelle fission”. In addition, GO terms related to energy utilization, such as “ATPase activity, coupled” and “ATPase activity”, were also included (Figure 5B). These enriched GO terms indicated the expression of *HpXCBP3* gene in OEH induced the changes of genes related to cell growth and proliferation, which is further revealed that *HpXCBP3* played roles on the regulation of cell growth and proliferation.

Based on the analysis of DEGs expression patterns between WT and OEH, 227 DEGs were grouped into 10 subclusters, with 172 up-regulated genes and 55 down-regulated genes (Figure 5C).

There were 60 genes in subcluster 1 and their expression patterns were up-regulated. GO terms indicates those gene were involved in DNA metabolic process, DNA replication, FACT complex, and cell division. In particular, three genes including CHLRE_06g293000v5 (large subunit of eukaryotic DNA polymerase), CHLRE_13g567700v5 (histone H2A), and CHLRE_02g104800v5 (histone H3) were up-regulated in OEH (Table S3). Moreover, the enrichment of GO terms including “cell cycle checkpoint” and “cell cycle process” indicated that the cell cycle process in OEH were also affected, showing up-regulation of CHLRE_08g372550v5 (type B cyclin-dependent kinase CDKB), CHLRE_08g370401v5 (type B cyclin CYCB,) and CHLRE_03g207900v5 (type A cyclin CYCA) (Table S3). The enrichment of GO terms including “SUMO ligase complex” and “ubiquitin-protein transferase activator activity” indicated that the ubiquitination was also affected. Among them, the expression of CHLRE_03g161500v5 gene, annotated as E3 ubiquitin ligase associating with the sensitivity of plants to salt stress, was up-regulated (Table S3). 

There were 57 genes in subcluster 2 and their expression patterns were up-regulated. The enrichment of GO terms such as “glutamate biological process”, “nitrogen utilization”, and “photochromogenesis”, indicated that the physiological processes related to chlorophyll synthesis and nitrogen utilization were active. The expression of CHLRE_13g592200v5, annotated as glutamate synthase, was up-regulated (Table S3). In addition, there were 27 genes in subcluster 4 and their expressions were up-regulated. The enriched GO terms also included “regulation of nitrogen compound metabolic process” and “heterocycle metabolic process”, which might be related to chlorophyll metabolism.

Specially, among DGEs, five WD40 genes including CHLRE_01g036100v5, CHLRE_02g073650v5, CHLRE_02g115250v5, CHLRE_06g255950v5, and CHLRE_16g652950v5 were up-regulated (Table S3). Among them, several genes had got GO annotations: CHLRE_02g073650v5 (GO:0016567 and GO:0004842), CHLRE_06g255950v5 (GO:0034388 and GO:0032040) and CHLRE_16g652950v5 (GO:0050662, GO:0004497 and GO:0003676). By querying the GO annotation above in THE GENE ONTOLOGY RESOURCE, it could be found that these genes are related to cell aging and CHLRE_02g073650v5 was also related to protein ubiquitination. Meanwhile, one TOC1 gene (CHLRE_16g676421v5) related to the regulation of circadian rhythm was also up-regulated (Table S3).

In brief, GO enrichment analysis of DEGs revealed that those genes related to energy utilization, DNA replication, cell cycle regulation, nitrogen utilization, circadian rhythm, and ubiquitination, were up-regulated. Moreover, it was also found that the expression of five WD40 proteins were also up-regulated. The up-regulation of these DEGs might explain phenotypic changes in OEH.

#### 2.5.2. KEGG Pathway Significant Enrichment Analysis of DEGs

According to the KEGG database, 227 DEGs were classified into four functional “first category” consisting of “Metabolism”, “Genetic Information Processing”, “Cellular Processes” and “Organismal Systems”. They were classified into 13 pathways of “second category” (Figure 5D). Based on the KEGG pathway significant enrichment analysis, it was found that the expression of genes related to DNA replication and nitrogen metabolism pathways were dramatically changed in transcriptional level.

**Figure 6:**
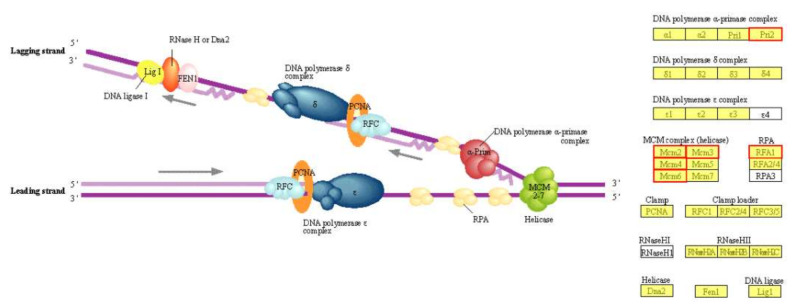
The pathways involved in DNA replication. The yellow background color indicates genes that exist in the reference genome. The red box indicates that gene expression is significantly up-regulated.

In the DNA replication process, the expression of six genes including the Pri2 subunit of DNA polymerase α-primase complex, the replication factor A1 (RFA1) of RPA, and four subunits of MCM complex were significantly increased (Figure 6, Tables S3 and S4). It could deduce that the active DNA replication process in OEH was one of the reasons for accelerating cell proliferation in the early growth stage.

**Figure 7:**
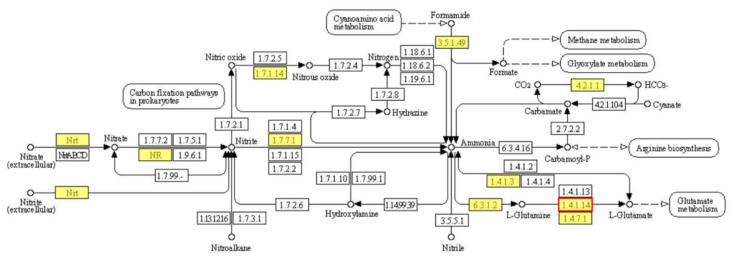
The pathways involved in nitrogen metabolism. The yellow background color indicates genes that exist in the reference genome. The red box indicates that gene expression is significantly up-regulated. The red box 1.4.1.14 is glutamate synthase (CHLRE_13g592200v5).

Even though increasing chlorophyll content was observed in OEH, the chlorophyll metabolism process (KEGG Pathway: map00860) did not undergo significant enrichment in the KEGG Pathway. Furthermore, genes involved in the process of chlorophyll synthesis were not significantly up-regulated. Further analysis indicated that the CHLRE_13g592200v5 gene, which was annotated as NADH-glutamate synthase, was significantly up-regulated. NADH-glutamate synthase participated in the nitrogen metabolism pathway (KEGG Pathway: map00910) and directly catalyzed the production of l-glutamate which was the substrate of chlorophyll biosynthesis (Figure 7, Tables S3 and S4). Therefore, it could be concluded that the up-regulation of CHLRE_13g592200v5 gene might cause the increased chlorophyll content in OEH.

## 3. Discussion

PLCPs participate in a variety of physiological activities in plants, such as growth, development, senescence, and stress response. Unfortunately, it is rarely reported the existence or the roles of PLCPs in microalgae, hindering the research on the mechanism of *H. pluvialis* responding to environmental stresses. In this study, a PLCP gene from *H. pluvialis*, named as *HpXBCP3*, was obtained. The deduced amino acids analysis showed that it contained the autoinhibitory prodomain, the protease domain, and the granulin domain, similar to DR21A and XBCP3 from *Arabidopsis*. The transcript level analysis indicated that *HpXBCP3* responded to NaCl stress in *H. pluvialis*. The *C. reinhardtii* transformants (OEH) expressing *HpXBCP3* performed differently from wide types (WT), including abnormal growth, higher sensitivity to NaCl stress, and improved chlorophyll content. Finally, transcriptome data indicated that DEGs related to cell cycle, DNA replication, ubiquitination, and nitrogen metabolism were up-regulated, suggesting that the *HpXBCP3* was involved in the growth and stress response in microalgae.

### 3.1. Expression of HpXBCP3 in C. reinhardtii Caused Abnormal Growth

Previous studies showed that PLCPs is closely related to plant growth and development. The growth of *Arabidopsis* mutants without functional *AtCEP1* was slowed down [7]. In this *cep1* mutant, the cell proliferation was delayed during early inflorescence development by affecting the expression of some genes during meiosis in anther cells [11]. The cell proliferation of microalgae *C. reinhardtii* was also similar to that of plants, which was controlled by cell cycle regulation and DNA replication [12]. It was well known that the cell cycle is regulated by cyclin-dependent kinase (CDK), CDK activated kinase (CAK), and E2F transcription factor [13]. DNA replication is associated with DNA polymerase [14], nucleosome replication, MCM complex, and replication factor A [15,16].

In this study, it was found that the expression levels of CYCA, CYCB, CDKA, DNA polymerase, replication factor A1, four MCM subunit genes, histone H2A, and histone H3 genes in the OEH were significantly up-regulated. These genes directly affected the cell cycle regulation and DNA replication in transgenic *C. reinhardtii* [13,14,17], leading to accelerating cell proliferation in the early growth stage of OEH, which finally caused premature senescence. Hence, the expression of *HpXBCP3* caused abnormal growth in *C. reinhardtii* by regulating genes involved in the cell cycle regulation and DNA replication (Figure S1A and Table S4).

### 3.2. HpXBCP3 Responded to NaCl Stress in Microalgae

Facing environmental stresses, recycling of proteins by plant proteolysis is a primary strategy for plant survival. In higher plants, the expression of PLCPs were increased under multiple environmental stresses [18,19], to accelerate the senescence of plant leaves or enhance plant sensitivity to abiotic stress [5]. In this study, it was found that NaCl stress improved the expression of *HpXBCP3* in *H. pluvialis*. The sensitivity of transgenic *Chlamydomonas* to salt stress was increased lead to more cell death, which might be associated with the protein ubiquitination.

In plants, E3 ubiquitin ligases are involved in the regulation of plant growth and development, and abiotic stress response by regulating protein ubiquitination [20,21,22]. Depending on the protein characteristics, RING E3 ubiquitin ligase could enhance stress tolerance or reduce salt stress tolerance in plants [21]. For example, the *OsMAR1*, a RING E3 ubiquitin ligase in rice, was up-regulated in rice under salt stress. However, over-expression of *OsMAR1* in *Arabidopsis* resulted in lower tolerance to salt stress, indicating that *OsMAR1* acted as a negative regulator of salt stress [23]. In this study, the expression of E3 ubiquitin ligase (CHLRE_03g161500v5) was up-regulated in OEH, which might be a reason for the lower salt stress tolerance (Table S4).

It was reported that over-expression of TOC1, a key component of the circadian clocks belonging to CCT family, significantly reduced the drought tolerance in *Arabidopsis* [24]. Moreover, the biorhythm-related CCT family genes in *Medicago truncatula* responded to NaCl stress [25]. In plants, WD40 proteins were involved in plant development, response to stress and other physiological activities [26,27]. In addition, several WD40 proteins were found to be involved in the ubiquitination of cellular proteins [28]. Moreover, the WD40 protein regulated by the circadian rhythm was associated with salt stress response in rice [29]. In this study, one TOC1 gene (CHLRE_16g676421v5) and five WD40 protein genes (CHLRE_01g036100v5, CHLRE_02g073650v5, CHLRE_02g115250v5, CHLRE_06g255950v5, CHLRE_16g652950v5) were significantly up-regulated in the OEH with the increasing of NaCl stress sensitivity. It was worth noting that three WD40 (CHLRE_02g073650v5, CHLRE_06g255950v5 and CHLRE_16g652950v5) were predicted relating to cell aging and one WD40 (CHLRE_02g073650v5) was predicted participating in the protein ubiquitin process. It could be inferred that WD40 protein weakened the tolerance of transformants to salt stress by affecting cell senescence and protein ubiquitination. However, the relationships between TOC1 and WD40 proteins were still unclear (Figure S1B and Table S4).

### 3.3. Expression of HpXBCP3 Leaded to Increased Chlorophyll Content

In plants, PLCPs was reported to affect the turnover of chloroplast proteins and the transmission of nuclear retrograde signals, thereby regulating the expression of photosynthetic genes [8]. In *C. reinhardtii*, the utilization of nitrogen affects the biosynthesis of chlorophyll [30]. In plants, glutamate synthase plays important roles in the process of nitrogen assimilation and catalyzing the synthesis of glutamate from glutamine and 2-oxoglutarate. In photosynthetic plants, the synthesis of chlorophyll a/b generally starts from l-glutamate and, includes multiple steps and many enzymes [31,32]. There are two main ways to synthesize l-glutamate. The first one is the synthesis of glutamate catalyzed by glutamyl-tRNA synthetase and the other one is the synthesis of l-glutamine catalyzed by glutamate synthase [33]. Moreover, previous studies pointed out that magnesium chelatase is the key enzyme and important regulator for chlorophyll synthesis and ATP is required to participate in the catalytic reaction [33,34].

In recent research, it was found that the deletion or over-expression of PLCP genes such as *AtCEP1* and *HvPAP14* could induce the expression change of plant photosynthetic genes, based on the transcriptome analysis [7,11]. Moreover, it was reported that PLCPs in the cytoplasm affected the chlorophyll content [8]. In this study, the transgenic *C. reinhardtii* expressing *HpXBCP3* showed a phenotype with increased chlorophyll content, compared with wild type. The transcriptome data suggested that biological processes involved in the synthesis of glutamate and the utilization of nitrogen were active in OEH. Further analysis found that the NADH-dependent glutamate synthase gene (CHLRE_13g592200v5) among the up-regulation genes might cause the increased chlorophyll content in OEH (Figure S1C and Table S4). The Energy metabolism process in OEH was also active, which might affect the activity of magnesium chelatase. Therefore, the expression of *HpXBCP3* up-regulated NADH-dependent glutamate synthase and thereby lead to the increment of chlorophyll content in OEH. 

**Figure 8:**
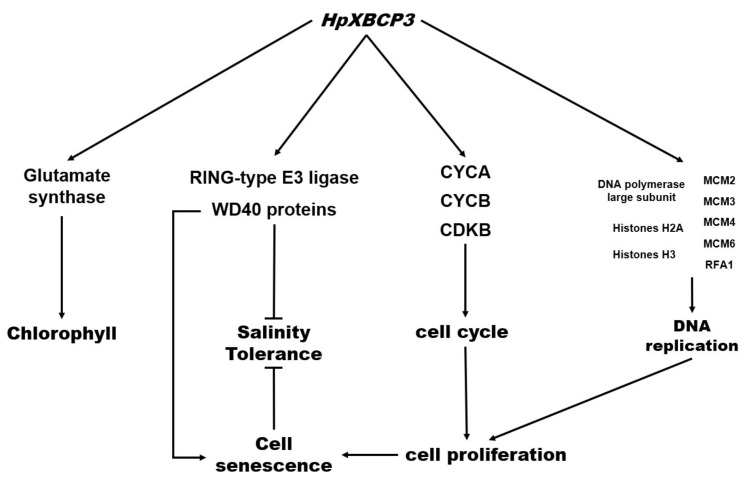
A model of the physiological effects of *HpXBCP3* in microalgae. Arrow indicates positive effects and blunt head indicates negative effects.

In this study, the *HpXBCP3* gene belonging to PLCPs was cloned from *H. pluvialis* and it responded to NaCl stress. *HpXBCP3* was then introduced to *C. reinhardtii* to study its functions. Several valuable results were obtained, including (1) *HpXBCP3* expression accelerated the growth in early culture phase, while caused premature senescence in *C. reinhardtii* by regulated the genes involved in cell cycle and DNA replication, (2) *HpXBCP3* expression increased the sensitivity to NaCl stress by regulating the ubiquitin pathway and WD40 proteins, and part of the WD40 proteins were also related to cell senescence, (3) *HpXBCP3* expression improved the chlorophyll content by up-regulating the expression of NADH-dependent glutamate synthases (Figure 8).

## 4. Materials and Methods

### 4.1. Algal Strains and Culture Conditions

The microalgae *H. pluvialis* strain 192.80 used in this study was obtained from the Sammlung von Algenkulturen Culture Collection of Algae (Göttingen, Germany) and algal cells were cultivated in ESP Ag medium (http://www.uni-goettingen.de/en/list-of-media-and-recipes/186449.html/ accessed on 26 October 2021) [35,36]. Cells were grown at 22 °C under continuous fluorescent light (30 μmol·m^−2^·s^−1^) in a growth chamber. After the growth reached to the logarithmic stage (about 1.5 × 10^5^ cell/mL), algal cells were treated with 86 mM NaCl [37]. Samples were taken at the time points of 0 h, 24 h, and 48 h, respectively. Cells were harvested by centrifugation at 6000× *g* for 5 min, immediately frozen in liquid nitrogen, and stored at −80 °C for further analysis.

The microalgae *Chlamydomonas reinhardtii* strain cc849 was a cell-wall-deficient mutant and purchased from the *Chlamydomonas* Genetic Center of Duke University (Duke University, Durham, NC, USA). The algal cells were cultured in a TAP (triglycol phosphate) liquid medium at 22 °C in an incubator with a continuous fluorescent light at the intensity of about 30 μmol·m^−2^·s^−1^. For the salt stress treatment, algae cells were collected at the logarithmic phase and resuspended to a final concentration of about 1.5 × 10^6^ cell·mL^−1^. Based on the literatures and our pretest results [38,39], the salt treatment was performed by adding NaCl to the final concentration of 300 mM into the medium for 24 h.

### 4.2. Microscope Observation and Growth Curve Determination

To evaluate the morphological changes of algal cells induced by NaCl treatment, algal cells were observed using an Olympus BX61 microscope and an Olympus DP10 digital camera (Olympus, Tokyo, Japan), at the time point of 0 h, 24 h, and 48 h after NaCl treatment. To compare the growth rate of the wild type and transgenic *C. reinhardtii*, algal cell densities were measured using a blood cell counting plate every day for a week and each sample was counted three times as replicates. The growth curve was constructed by linearizing the cell density with collection time.

### 4.3. Cloning of HpXBCP3 and Bioinformatic Analysis

To amplify the *HpXBCP3*, primers (Forward: 5′-TCGAACTCTCCAGAAACTCGT-3′ and Reverse: 5′-TCAAGCCACCACTTTGTCA-3′) were designed according to the full-length transcriptome sequencing data of *H. pluvialis*. Total RNA was extracted by RNA fast 200 Kit (Fastagen, Shanghai, China, www.fastagen.cn/ accessed on 26 October 2021) from algal cells of *H. pluvialis* grown under normal conditions. The cDNA synthesis was performed by PrimeScript™ RT reagent Kit with gDNA Eraser (Perfect Real Time) based on the user instruction (Takara, Beijing, China). The amplification reaction was carried out using Platinum™ SuperFi™ DNA Polymerase (Invitrogen™, Shanghai, China) according to the manufacturer’s protocol. The nucleotide sequence data of *HpXBCP3* was uploaded to NCBI database (accession number: MZ508431).

The ORF and amino acid sequences were analyzed by ORF finder online (https://www.ncbi.nlm.nih.gov/orffinder/ accessed on 26 October 2021). The sequence alignment and conservative domain prediction were performed by NCBI (https://blast.ncbi.nlm.nih.gov/Blast.cgi/ accessed on 26 October 2021). The phylogenetic tree was constructed using the Neighbor-joining method from MEGA X software (Institute for Genomics and Evolutionary Medicine, Temple University, Philadelphia, PA, USA). Multiple alignment of the PLCPs CDS sequence from different species was performed using DNAMAN 8.0 software (Lynnon Biosoft, San Ramon, CA, USA). Schematic of gene structure was carried out using GSDS 2.0 (http://gsds.gao-lab.org/ accessed on 26 October 2021). Subcellular localization of *HpXBCP3* was predicted by three independent software including YLOC (https://abi-services.informatik.uni-tuebingen.de/yloc/webloc.cgi?page=info/ accessed on 26 October 2021), Plant-Ploc (http://www.csbio.sjtu.edu.cn/bioinf/plant/ accessed on 26 October 2021), and PSORT (https://psort.hgc.jp/ accessed on 26 October 2021).

### 4.4. Detecting the Gene Expression Pattern Responding to Salt Stress by qRT-PCR

To investigate the *HpXBCP3* expression pattern under NaCl stress, qRT-PCR was performed on an ABI QuantStudio™ 6 Flex System (Applied Biosystems, Shanghai, China) using TB Green™ Premix Ex Taq ™II (Tli RNaseH Plus) (Takara, Beijing, China). The templates used for qRT-PCR were cDNA of algal cells collected at the time points of 0 h, 24 h, and 48 h after NaCl stress treatment. Primers used were qF (5′-ACGGTGCTTGAGGTGGTTAG-3′) and qR (5′-ACACGAAGTCCCTCGTTTCC-3′) for *HpXCBP3*. The amplification conditions included the initial denaturation of 95 °C for 30 s, then 40 cycles of 95 °C for 5 s followed by 60 °C for 30 s. The gene *HpActin*, which was amplified by primer qHpActinF: 5′-AGCGGGAGATAGTGCGGGACA-3′ and qHpActinR: 5′-ATGCCCACCGCCTCCATGC-3′, was used as the endogenous reference [40]. Expression levels of each gene was determined and calculated by 2^−ΔΔCt^ methods [41]. All reactions were run in triplicate and at least three biological replicates were used.

### 4.5. Plasmid Construction and Genetic Transformation

**Figure 9:**
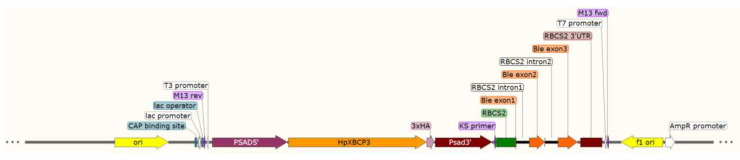
The sketch map of vectors used for *Chlamydomonas reinhardtii* transformation.

The expression vector pDb124, which was used for the microalgae transformation, contains the gene expression cassette using *PsaD* 5′ and *PsaD* 3′ UTR to express the target gene constitutively and the zeocin resistance to select the transformants [42]. In recent years, the most commonly used expression elements in *C. reinhardtii* were the promoter and terminator from the photosystem I reaction center subunit II (PSAD) and ribulose bisphosphate carboxylase small subunit 2 (RBCS2) promoters [43], which could efficiently and reliably express target gene in *Chlamydomonas*. The CDS of *HpXBCP3* together with 3 × HA tag at the 5’ end was synthesized by Sangon Biotech (https://www.sangon.com/ accessed on 26 October 2021) and then incorporating it into the pDb124 vector between *PsaD* 5′ and *PsaD* 3′ UTR to obtain the plasmid pDb124-HpXBCP3. The main structure of plasmid vector was shown in Figure 9.

Following the protocol previously described using glass beads method [42,44], the algal cells were cultivated in 100 mL TAP liquid medium in an incubator at 22 °C with continuous light intensity of 30 μE·m^−2^·s^−1^ until the cell density reached to 1~2 × 10^6^ cell/mL. Algal cells were harvested by centrifugation at 4500× *g* for 5 min and resolved with 300 μL TAP liquid medium to keep the final density of 2 × 10^8^ cell/mL. Cell cultures were transferred to a new 1.5 mL microcentrifuge tube containing 300 mg glass beads (0.5 mm in diameter) and about 1.5 μg linearized pDb124-HpXBCP3 plasmid. The glass beads were blown gently and vortexed at the speed of 2500 rpm for 25 s. Then, the supernatant was transferred to a new microcentrifuge tube with 10 mL TAP liquid medium and shaken continuously at 100 rpm for about 24 h at 22 °C with a light intensity of 30 μE·m^−2^·s^−1^. Ultimately, cells were harvested by centrifugation at 3000× *g* for 5 min, resolved with 100 μL TAP liquid medium, and spread on the TAP agar medium containing 10 μg/mL zeocin and 100 μg/mL ampicillin. These algal cells were cultivated at 22 °C in an incubator under a continuous light intensity of 30 μE·m^−2^·s^−1^ for about 2–3 weeks until the green colonies were visible.

Transgenic algal strains screened by antibiotics were verified by PCR using genomic DNA with the primer F: 5′-ACTGGACCGTGAAGAACAGC-3′ and the primer R:5′-CCGAGCAAACAGACAGTCCA-3′ targeting *HpXBCP3* gene (485 bp). Ultra DNA isolation kit was used for DNA isolation (BEI-BEI BIOTECH, Zhengzhou, China, http://www.beibeibio.com/ accessed on 26 October 2021). To test the expression of *HpXBCP3* in transformants confirmed by PCR using genomic DNA, RT-PCR was performed. Total RNA of transformants was extracted and RT-PCR was carried out with the primer F: 5′-CCGTCAAGTCTGGTCTGTGT-3′ and the primer R: 5′-CCCAGTAGCTACGGTGGTTC-3′ targeting *HpXBCP3* gene (295 bp). All PCR reactions were conducted as using 2 × M5 HiPer Taq HiFi PCR mix (Mei5 Biotechnology Co., Ltd., Beijing, China) following the manufacturer’s instructions PCR products were analyzed by 1% agarose gel electrophoresis.

### 4.6. Determination of Chlorophyll and Carotenoids Content

The chlorophyll and carotenoids content were determined to evaluate the phenotypic changes of transformants. As described by Lin et al. [45], for each sample, algae cells were harvested by centrifugation at 12,000× *g* for 5 min at 4 °C. The pellet was washed by ddH_2_O and dried using a freeze-dryer. The pellet was crashed into powder weighted, and re-suspended in 1 mL of 80% acetone. After incubation at 55 °C for 30 min, the acetone extract was centrifuged at 12,000× *g* for 5 min at 4 °C. The supernatant was collected and the absorbance was detected at 470, 645, and 663 nm using a BioTek Instruments spectrophotometer. The contents of chlorophyll and carotenoids per mL of algal samples were estimated using the following equations: chlorophyll a (μg·mL^−1^) = 12.7 (A663) − 2.69 (A645), chlorophyll b (μg·mL^−1^) = 22.9 (A645) − 4.68 (A663) and carotenoids (μg·mL^−1^) = [1000 (A470) – 3.27 Ca – 104 Cb]/229. Finally, the contents of chlorophyll data were normalized per milligram of dry weight.

### 4.7. Transcriptomic Analyses

When the wild-type of *C. reinhardtii* cc849 and two transgenic *Chlamydomonas* lines (H12 and H28) grew to the middle of logarithmic phase, a sufficient (not less than 1 × 10^−7^ cells) amount of algae culture was collected, immediately frozen in liquid nitrogen, and stored at −80 °C. Two transgenic *Chlamydomonas* lines (H12 and H28) were randomly selected from positive transformants showing high expression of *HpXBCP3*. Samples were stored in dry ice and sent to Majorbio (http://www.majorbio.com/ accessed on 26 October 2021) for high throughput RNA sequencing. Each sample had two biological replicates.

Transcriptome sequencing including RNA extraction, library construction, sequencing, sequence assembly, and annotation were completed by Majorbio (http://www.majorbio.com/ accessed on 26 October 2021). Briefly, raw reads were processed using FastX—Toolkit, and reads containing ploy-N and the low-quality reads were removed by SeqPrep and Sickle to obtain clean reads, which were then mapped to the *C. reinhardtii* reference genome (reference genome version: Chlamydomonas_reinhardtii_v5.5; source of reference genome: http://plants.ensembl.org/Chlamydomonas_reinhardtii/Info/Index?db=core/ accessed on 26 October 2021) using TopHat2 [46].

Cufflinks (2010) was used to calculate the Fragments Per Kilobases per Millionreads (FPKM) value and HTSeq package read was used to count each gene. Differentially expressed genes (DEGs) were identified using DEGSeq, with *p*-adjust < 0.05 and |log2FC| > = 1 as the threshold to demonstrate the significance of differential expression. Gene ontology (GO) enrichment analysis of DEGs was performed using Goatools. GO terms with a corrected *p*-value < 0.05 were considered as significantly enriched among DEGs. Cluster analysis of gene expression patterns was implemented based on K-means method [47]. KEGG pathway enrichment analysis of DEGs was performed using the R scripts, calculation principle and significance analysis method following GO functional enrichment analysis. All these analyses mentioned above were performed based on the integrated cloud platform in I-Sanger (https://www.i-sanger.com/ accessed on 26 October 2021). The methodology data analysis and other specific information were available on this website.

To verify the accuracy of DEGs identification, the expression of eight DEGs selected based on fold changes and/or functional relevance was experimental confirmed by qRT-PCR. Endogenous reference gene *CrActin* was used as the internal control to normalize the expression data and relative gene expression level was calculated using the 2^–ΔΔCt^ method [48]. The qRT-PCR was accomplished as described above and primers were shown in Table 2.

The transcriptome data was deposited in the National Center for Biotechnology Information Gene Expression Omnibus database under accession number PRJNA743800.

### 4.8. Statistical Analysis

Data in figures and tables were shown as the average of at least three biological replicates with standard deviation. *t*-test (GraphPad Prism 5.0/8.0) was performed for statistically analysis, and *p*-value of <0.05 were considered as statistical significances.

## 5. Conclusions

This study firstly reported a PLCP gene *HpXBCP3* from *H. pluvialis* and its biological functions. Our studies elucidated that *HpXBCP3* might involve in regulating the growth, stress response, and chlorophyll synthesis in microalgae. These results would help to further understand the functions of PLCPs and deepen the understanding of the response mechanism to environmental stresses in *H. pluvialis*.

## Figures and Tables

**Table 1:** Subcellular location prediction of *HpXBCP3*.

Online Analysis Software	Predicted Location(s)
YLOC	Cytoplasm
Plant-PLoc	Cytoplasm
PSORT	Cytoplasm/Chloroplast stroma

**Table 2:** Nucleotides of primers used in qRT-PCR.

Gene ID	Forward Primer 5′-3′	Reverse Primer 5′-3′
CHLRE_01g019250v5	GGGTCACTACATCGTGGCTT	TGATGACGGAGTGGTTGGAC
CHLRE_08g372550v5	AGCAGCTATGGATGCCTACG	TTCAAGGCGGCACTTCTTCA
CHLRE_16g656700v5	CGCAACTCCAAGCTCACCTA	TTTCGCACGCATTGACCTTG
CHLRE_13g603350v5	GTCAGCGTCCCAACAAAAACA	ACCTTGTACACACCGAACGC
CHLRE_01g019250v5	GTCCAACCACTCCGTCATCA	ATCCAGCTGCTTGTACTCGG
CHLRE_01g045200v5	CGAGACCAACCAGACGAAGT	GTAGTTCAGCATCCAGCCGT
CHLRE_12g491050v5	CACTCAGCTCATGTCTCCTCC	ATAGGGAACATGCAGAAGCGG
CHLRE_12g558100v5	CCGACTTCGCCAACTACTTCT	CCACCGCGTACACCTTCTT
*CrActin*	ACCCCGTGCTGCTGACTG	ACGTTGAAGGTCTCGAACA

## Data Availability

Not applicable.

## Supplementary Materials

The following are available online at https://www.mdpi.com/article/10.3390/ijms222111539/s1.
