# Supplementary material for: The Papain-like Cysteine Protease HpXBCP3 from Haematococcus pluvialis Involved in the Regulation of Growth, Salt Stress Tolerance and Chlorophyll Synthesis in Microalgae
Source: Int J Mol Sci. 2021 Oct 26;22(21):11539. doi: 10.3390/ijms222111539 (PMC8583958; doi:10.3390/ijms222111539)
Supplement: Supplementary file 1 [file ijms-22-11539-s001.zip › ijms-1364156-supplementary.pdf]

Table S1. Accession number of *CrCEP1* and *Arabidopsis* PLCPs in NCBI.

Gene Name	Accession No. in NCBI
AtALP	At5g60360
AtALP2	At3g45310
AtCEP1	At5g50260
AtCEP2	At3g48340
AtCEP3	At3g48350
AtCTB1	At1g02300
AtCTB2	At1g02305
AtCTB3	At4g01610
AtPAP1	At2g34080
AtPAP2	At1g29090
AtPAP3	At1g29080
AtPAP4	At2g27420
AtPAP5	At3g49340
AtRD19A	At4g39090
AtRD19B	At2g21430
AtRD19C	At4g16190
AtRD19D	At3g54940
AtRD21A	At1g47128
AtRD21B	At5g43060
AtRD21C	At3g19390
AtRDL1	At4g36880
AtRDL2	At3g19400
AtRDL3	At3g43960
AtRDL4	At4g11310
AtRDL5	At3g11320
AtRDL6	At4g23520
AtSAG12	At5g45890
AtTHI1	At1g06260
AtXBCP3	At1g09850
AtXCP1	At4g35350
AtXCP2	At1g20850
CrCEP1	XM_001697570.1

Table S2. Comparison of expression levels of 8 genes obtained by qRT-PCR analysis and by RNA-seq.

	H12 vs WT		H28 vs WT	
gene ID	Transcriptomic data	qRT-PCR	Transcriptomic data	qRT-PCR
CHLRE_01g019250v5	2.241293166	1.537941	2.741623102	2.624
CHLRE_08g372550v5	2.153336356	3.483752	4.247394795	2.099
CHLRE_16g65	2.255848981	1.435063	3.340206019	1.739001

6700v5				
CHLRE_13g603350v5	0.189475239	0.870019	0.233921914	0.359051
CHLRE_01g019250v5	2.241293166	1.495483	2.741623102	1.429616
CHLRE_01g045200v5	0.237564735	0.071814	0.370577869	0.055956
CHLRE_12g491050v5	2.088139141	1.278584	3.328684036	2.197714
CHLRE_12g558100v5	2.05829569	1.406596	2.281356788	3.902872
Pearson Correlation Coefficient		0.6637		0.9655

Table S3. The annotations of genes involved in this article in Genebank and Phytozome. Blast the selected sequences in the transcriptome data in genebank (https://www.ncbi.nlm.nih.gov/genbank/) and phytozome (https://phytozome-next.jgi.doe.gov/) to find the sequences with the highest similarity, and the annotation results are consistent with the annotations in the transcriptome data.

Definition	gene_id	Accession number in genebank	E-value	Accession number in Phytozome	E-value
Pri2	CHLRE_06g293000v5	XM_001701548.1	0	Cre06.g293000.t1.1	0
RFA1	CHLRE_16g651000v5	XM_001698105.1	0	Cre16.g651000.t1.2	0
MCM2	CHLRE_07g338000v5	XM_001700028.1	0	Cre07.g338000.t1.2	0
MCM3	CHLRE_06g295700v5	XM_001691274.1	0	Cre06.g295700.t1.1	0
MCM4	CHLRE_07g316850v5	XM_001700758.1	0	Cre07.g316850.t1.2	0
MCM6	CHLRE_03g178650v5	XM_001703203.1	0	Cre03.g178650.t1.1	0
Histone 2A	CHLRE_13g567700v5	XM_001693648.1	0	Cre13.g567700.t1.2	0
Histone H3	CHLRE_02g104800v5	XM_001699933.1	2.00E-162	Cre02.g104800.t1.1	0
CDKB1	CHLRE_08g372550v5	XM_001701247.1	0	Cre08.g372550.t1.1	0
CYCA1	CHLRE_03g207900v5	XM_001693115.1	0	Cre03.g207900.t1.1	0
CYCB1	CHLRE_08g370401v5	XM_001701235.1	0	Cre08.g370401.t1.1	0
RING E3	CHLRE_03	XM_001703509	0	Cre03.g161500.t	0

ubiquitin ligase	g161500v5	.1		1.1	
Glutamate synthase (NADH)	CHLRE_13 g592200v5	XM_001693030 .1	0	Cre13.g592200.t 1.2	0
WD40 protein	CHLRE_01 g036100v5	XM_001689586 .1	0	Cre01.g036100.t 1.1	0
	CHLRE_02 g073650v5	XM_001701768 .1	0	Cre02.g073650.t 1.2	0
	CHLRE_02 g115250v5	XM_001699601 .1	0	Cre02.g115250.t 1.1	0
	CHLRE_06 g255950v5	XM_001696319 .1	0	Cre06.g255950.t 1.1	0
	CHLRE_16 g652950v5	XM_001698168 .1	0	Cre16.g652950.t 1.2	0
TOC1, APRR1	CHLRE_16 g676421v5	XM_001695726 .1	0	Cre16.g676421.t 1.1	0

Table S4. The DEGs involved in this study and their relative expression level in OEH to WT.

Biological process	gene_id	Definition	OEH VS WT
DNA replication	CHLRE_06g293000v5	Pri2	up 2.33
	CHLRE_16g651000v5	RFA1	up 2.71
	CHLRE_07g338000v5	MCM2	up 3.87
	CHLRE_06g295700v5	MCM3	up 3.28
	CHLRE_07g316850v5	MCM4	up 3.70
	CHLRE_03g178650v5	MCM6	up 4.33
	CHLRE_13g567700v5	Histone 2A	up 3.63
	CHLRE_02g104800v5	Histone H3	up 3.56
cell cycle regulation	CHLRE_08g372550v5	CDKB1	up 4.25
	CHLRE_03g207900v5	CYCA1	up 3.41
	CHLRE_08g370401v5	CYCB1	up 3.42
protein ubiquitination	CHLRE_03g161500v5	RING E3 ubiquitin ligase	up 3.77

	CHLRE_02g073650v5	WD40 protein	up 3.34
cell senescence	CHLRE_06g255950v5		up 3.19
	CHLRE_16g652950v5		up 3.78
	CHLRE_01g036100v5		up 2.93
	CHLRE_02g115250v5		up 3.80
	CHLRE_16g676421v5	TOC1, APRR1	up 2.89
NADH-dependent glutamate synthase	CHLRE_13g592200v5	Glutamate synthase (NADH)	up 3.22

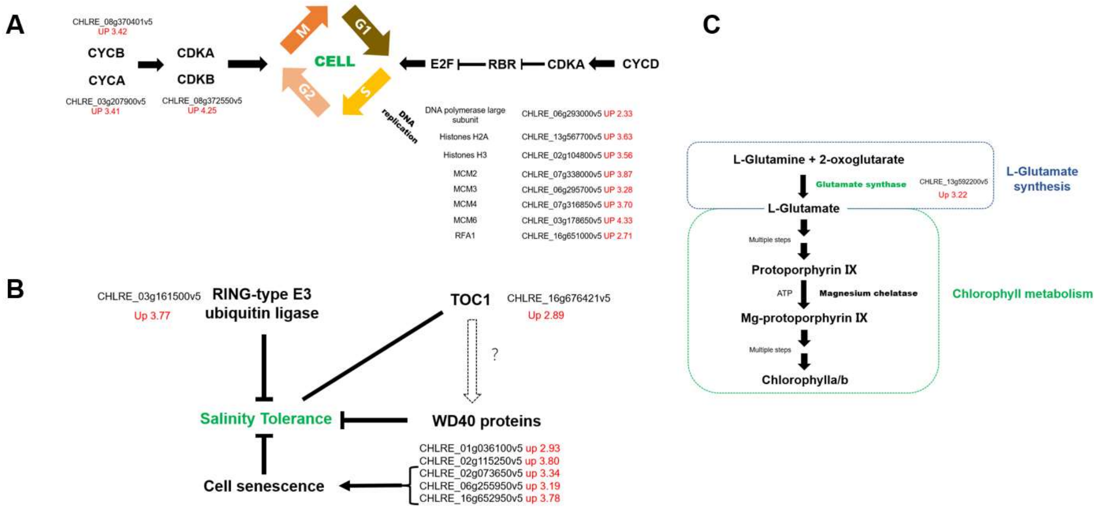

Figure S1. (A) Genes involved in cell cycle regulation and DNA replication were up-regulated in OEH. The fold changes of genes were in red. (B) Up-regulation of RING-type E3 ligase and WD 40 proteins decreased salt stress tolerance in OEH. Arrow indicates positive effects and blunt head indicates a positive effect. The straight line indicates a relationship. The dotted arrow indicates that the relationship is unknown. The fold changes of genes were in red. (C) Chlorophyll synthesis affected by *HpXBCP3* expression in OEH.
